# Plasma aldosterone response to ACTH stimulation test for diagnosis of primary aldosteronism: a cross-sectional study

**DOI:** 10.1186/s12902-024-01563-y

**Published:** 2024-03-13

**Authors:** Worapaka Manosroi, Pichitchai Atthakomol, Piti Inthaphan, Supornthip Hintong

**Affiliations:** 1 Division of Endocrinology, Department of Internal Medicine, Faculty of Medicine, Chiang Mai University, 110 Intrawarorot Road Soi 2, Si Phum, Amphoe Mueang Chiang Mai, Chiang Mai, 50200 Chiang Mai, Thailand; 2 Clinical Epidemiology and Clinical Statistic Center, Faculty of Medicine, Chiang Mai University, Chiang Mai, Thailand; 3 Orthopaedics Department, Faculty of Medicine, Chiang Mai University, Muang Chiang Mai, Chiang Mai, Thailand; 4 Department of Internal Medicine, Nakornping Hospital, Chiang Mai, Thailand

**Keywords:** Primary aldosteronism, ACTH stimulation test, Serum aldosterone, Serum cortisol

## Abstract

**Background:**

The diagnosis of primary aldosteronism (PA) requires screening and confirmation testing. The present study examined whether the 1 µg ACTH stimulation test for plasma aldosterone concentration (PAC) can accurately diagnose PA by bypassing the regular confirmatory steps of PA diagnosis.

**Methods:**

A cross-sectional study with a total of 36 patients with an aldosterone-renin ratio (ARR) > 20 ng/dL per ng/m/hr were included. The confirmation test for PA was performed by saline infusion and the patients were categorized into PA and non-PA. PAC was collected at 20 and 40 min after 1 µg ACTH stimulation test. Multivariable logistic regression analysis was performed, and the associations are presented as odds ratios (OR) and 95% confidence intervals (CI). Diagnostic accuracy is presented as AuROC.

**Results:**

Multivariable analysis found only PAC at 20 min after ACTH stimulation showed significant association with a diagnosis of PA (OR 1.18, 95%CI (0.99, 1.31), *p* = 0.040). AuROC for this value was 0.95 and the proposed cut-off was 52 ng/dL with a sensitivity of 71.4% and a specificity of 96.6%.

**Conclusions:**

Diagnosing PA may be aided by PAC at 20 min following 1 µg ACTH stimulation. This value may be used with patients for whom the confirmation test for PA cannot be conducted.

**Supplementary Information:**

The online version contains supplementary material available at 10.1186/s12902-024-01563-y.

## Introduction

Primary aldosteronism (PA) is the most common cause of secondary hypertension, accounting for 60% of resistant hypertension cases [1]. According to current guidelines, the diagnosis of PA is comprised of a screening test and a confirmatory test [2]. The screening test includes measuring the aldosterone-renin ratio (ARR) which is calculated from the plasma aldosterone concentration (PAC) and plasma renin activity (PRA). If ARR is higher than the suggested cut-off level, confirmation tests should be conducted. The confirmation tests are the saline infusion test (SIT), captopril challenge test, fludrocortisone suppression test and oral sodium loading test. Among these, SIT is the most commonly used test for confirmation of PA [3]. Although the cost of performing this test is low, some potentially adverse events can occur following SIT including increased blood pressure, volume overload, and low potassium levels [4].

Angiotensin II plays a major role in aldosterone secretion from the zona glomerulosa of the adrenal gland. In addition to angiotensin II, ACTH and potassium can also regulate aldosterone synthesis [5]. The ACTH stimulation test, which is normally used to diagnose adrenal insufficiency, has been reported to be helpful in differentiating between PA and low-renin hypertension [6]. One study demonstrated that the 250 µg ACTH stimulation test can help differentiate subtypes of PA with a high degree of accuracy in the diagnosis of aldosterone-producing adenoma (APA) [7]. Combined with abdominal computed tomography (CT), the 250 µg ACTH stimulation test can also differentiate PA subtypes [8]. Also, some studies have reported conflicting results. Two studies reported that PAC after a 250 µg ACTH stimulation test does not facilitate the confirmation of PA while only one study stated that this test can confirm PA [9–11].

All of the above-mentioned studies used a high (250 µg) ACTH stimulation test which is a supramaximal dose for adrenal gland stimulation. In order to mimic a physiologic adrenal response, some experts have suggested the use of a low dose ACTH stimulation test with more frequent blood sampling of aldosterone levels [12]. Only one study was performed using a 1 µg ACTH stimulation test to differentiate APA from other functioning adrenal tumors [13]. There have been no studies of a 1 µg ACTH stimulation test to facilitate the diagnosis of PA.

The aim of the present study was to examine whether a low dose (1 µg) ACTH stimulation test for PAC can help diagnose PA while bypassing the confirmation step of PA diagnosis. We also determined the optimal cut-off value for significant PAC results after a low dose ACTH stimulation test.

## Materials and methods

This cross-sectional study was conducted at the outpatient endocrine and metabolism unit of a tertiary medical center between March and October 2021. The study was approved by the Faculty of Medicine, Chiang Mai University Ethical Committee (355/2562). The study adhered to the Standards for Reporting of Diagnostic Accuracy Studies (STARD) guideline (Supplementary Appendix). All methods were carried out in accordance with declaration of Helsinki. Written informed consent was obtained from all participants before participating in the study. All subjects were evaluated and screened for study eligibility by the first author (WM) prior to study entry. A total of 36 adult patients aged over 18 years with an ARR > 20 ng/dL per ng/mL/hr and who had indications suggesting investigation for PA according to the current PA Guidelines were included in this study [2, 14]. The indications were comprised of resistant or severe hypertension, hypertension with hypokalemia, hypertension in patients under the age of 40, hypertension with adrenal incidentaloma or atrial fibrillation or sleep apnea and a family history of early onset hypertension or of cerebrovascular accident at age < 40 years. Exclusion criteria were pregnancy, a history of Cushing’s syndrome, adrenal insufficiency, chronic kidney disease (eGFR < 30 mL/min/1.73m^2^), heart failure, metastatic cancer, current use of glucocorticoids and a history of ACTH allergy. The information from the participants can be identified by the research team but is not revealed to anybody outside the research project. The data that supports the findings of this study are available upon request from the authors.

### The ACTH stimulation test for plasma aldosterone concentration and serum cortisol

The ACTH stimulation test was performed at an outpatient clinic between 8:00 a.m. and 9:00 a.m. by medical nurses. The patients’ potassium levels must be greater than 3.5 mEq/L. An intravenous cannula was inserted into a forearm vein, and the patients were rested for 15 min. before starting the test. First, serum basal cortisol and PAC at 0 min were determined. That was followed by 1 µg ACTH administered intravenously, after which serum cortisol and PAC were determined at 20 and 40 min. Serum cortisol was collected to be used for the adjustment of PAC levels. The 1 µg ACTH ampules were prepared by pharmacists under sterile conditions by diluting the 250 µg ampules of ACTH with normal saline, which were then stored at 2–8 °C. Ampules were used within 60 days after dilution [15, 16]. 

### Assay methods

PAC assay was accomplished using direct ELISA assays (DiaMetra Ekit, Spello, Italy) with a reference range in the upright position of 3–40 ng/dL and in the supine position of 2–18 ng/dL. The intra-assay variability was < 9.7%, and the inter-assay variability was < 11%. PRA was determined using a direct ELISA assay (DRG Instruments GmBH, Germany) with a reference range of 0.06–4.69 ng/mL/hr. Serum cortisol levels were measured by electrochemiluminescence assay (ECLIA) (Elecsys®, Cortisol II assay, Roche Diagnostics GmbH, Mannheim, Germany).

### Diagnosis procedure for primary aldosteronism

Screening for PA was performed according to the Endocrine Society guideline 2016 [2]. In brief, PAC and PRA were collected between 7:00 a.m. and 9:00 a.m. The patients’ potassium levels must be greater than 3.5 mEq/L. Patients taking beta-blockers, ACE inhibitors, or ARBs were required to stop taking them at least two weeks before the screening test. Patients taking diuretics and drugs that block mineralocorticoid receptors were instructed to stop taking them at least 4 weeks before the screening test. For the treatment of hypertension, only slow-release verapamil, hydralazine, and/or alpha-blockers were permitted to be continued. Samples for PAC and PRA analysis were drawn from patients in the upright position after they had been seated for 5–15 min. Patients who underwent the saline suppression test for PA confirmation had an upright ARR > 20 ng/dL per ng/mL/hr and suppressed PRA. The confirmation test consisted of infusing 0.9% normal saline at a rate of 500 mL/hr for 4 h while sitting. After the infusion was completed, PAC was assessed. Patients with PAC levels more than 6 ng/mL were diagnosed as having PA.

### Definitions

Non-PA patients were those with a negative confirmation test for PA. 20- and 40-minute delta PAC were defined as the difference between the value of PAC before the ACTH stimulation test (0 min) and the PAC level at 20 and at 40 min after the ACTH stimulation test, respectively. Hypertension in the young was defined as patients < 40 years who were diagnosed with hypertension. The results of PAC after the ACTH stimulation test and the confirmed result of PA were not blinded to the assessor.

### Statistical analysis

For statistical analysis, the STATA program (Stata Corp., College Station, Texas, USA) was employed. Counts or percentages are reported for categorical data, whereas means and standard deviation (SD) are shown for continuously distributed variables with a normal distribution. Interquartile ranges (IQR) are presented for continuous variables with a non-normal distribution. For continuous data, the Wilcoxon rank-sum test was used for non-normally distributed variables and the independent t-test for normally distributed variables. To evaluate the relationship between PAC following an ACTH stimulation test and PA diagnosis, multivariable logistic regression analysis was employed. The data is displayed as unadjusted and adjusted odds ratios (ORs) and 95% confidence intervals (CI). The model was adjusted for age, sex and body mass index (BMI). Area under the receiver operating characteristics (AuROC) and the selection of optimal cut-off values were performed only for values which demonstrated statistically significant results in multivariable analysis. The optimal cut-off value was selected by Liu index [17]. Statistical significance was set at *p* < 0.05. The sample size calculation was not performed due to this was a pilot study. Missing data and inclusive results were excluded from the study.

## Results

### Baseline characteristics

**Fig. 1:**
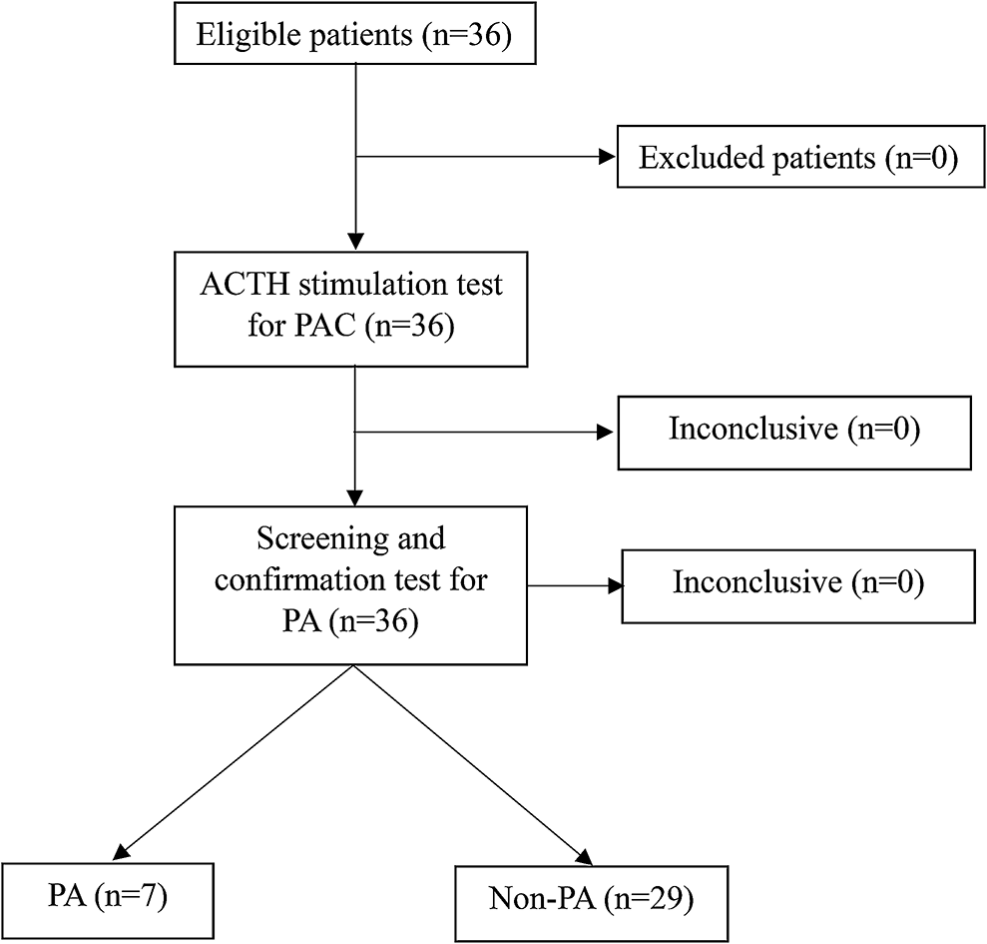
AuROC of plasma aldosterone concentration 20 min after ACTH stimulation test

Among the 36 patients, 7 had PA while 29 were non-PA. The mean age of the patients was 33.0 ± 11.7 years; 69% were male. Those with PA were significantly older than those with non-PA. The most common comorbidity in the included subjects was dyslipidemia (36%). The most common indication for PA screening was hypertension in young patients at 80.6%. PAC levels were significantly higher in the PA than in the non-PA group, while there was no significant difference in PRA between the groups. ARR were significantly higher in PA than in non-PA patients. The approximate time interval between the PA confirmation test and the ACTH stimulation test was 1 month, and there was no clinical intervention during this time interval. No adverse effect occurred after PA screening and confirmation tests in all patients. The baseline characteristics of the PA patients and non-PA groups are presented in Table 1 and flow diagram of the study is shown in Fig. 1.

### ACTH stimulation test for plasma aldosterone concentration

PAC levels at 20 and 40 min and at maximum PAC after the ACTH stimulation test were significantly higher in PA patients than in non-PA patients (*p* < 0.005, *p* < 0.005 and *p* < 0.014, respectively). Maximum PAC adjusted by serum cortisol for the same time period (maximum PAC/cortisol) showed significantly higher levels in PA than in non-PA patients (*p* < 0.005). Changes in PAC after the ACTH stimulation test (the 20- and 40-minute delta PAC) were significantly higher in PA than in non-PA patients (*p* < 0.005 and *p* < 0.005, respectively). No adverse effect occurred after ACTH stimulation test in all patients. The results are as shown in Table 1.

**Table 1:** Baseline characteristics

Baseline characteristics	Primary aldosteronism (n = 7)	Non-primary hyperaldosteronism (n = 29)	p-value
Male, n (%)	5 (71.4)	20 (68.9)	0.899
Age, mean ± SD (years)	46.2 ± 16.1	29.8 ± 7.8	< 0.005
Body mass index, mean ± SD (kg/m^2^)	27.8 ± 1.6	30.9 ± 6.0	0.193
Systolic blood pressure, mean ± SD (mmHg)	143.5 ± 5.4	139.6 ± 2.8	0.537
Diastolic blood pressure, mean ± SD (mmHg)	93.1 ± 11.9	87.1 ± 21.1	0.479
Underlying disease, n (%) - Diabetes mellitus - Stroke - Atrial fibrillation - Dyslipidemia	0 (0) 0 (0) 1 (14.3) 3 (42.8)	3 (10.3) 1 (3.5) 1 (3.4) 10 (34.5)	0.374 0.618 0.261 0.679
Smoker, n (%)	2 (28.6)	2 (6.9)	0.101
Duration of hypertension, median (IQR) (months)	36 (6, 120)	12 (7, 36)	0.149
Indication for testing, n (%) - Hypokalemia	4 (57.1)	2 (6.9)	< 0.005
- Hypertension in the young	3 (42.8)	26 (89.6)	0.005
- Adrenal incidentaloma	1 (14.3)	0 (0)	0.039
- Uncontrolled hypertension	1 (14.3)	0 (0)	0.039
- Obstructive sleep apnea	0 (0)	5 (17.2)	0.236
Potassium level, mean ± SD (mEq/L)	3.6 ± 0.1	3.6 ± 0.1	0.830
PAC, median (IQR) (ng/dL)	17.6 (10.4, 24.9)	9.9 (6.1, 14.9)	0.056
PRA, median (IQR) (ng/mL/hr)	0.2 (0.06, 0.77)	0.8 (0.38, 1.61)	0.206
Ratio of PAC to PRA, median (IQR) (ng/dL per ng/(mL·hr))	52.4 (28.4, 500.3)	28.1 (21.9, 35.8)	< 0.005
**Plasma aldosterone concentration before and after 1 µg ACTH stimulation test**			
PAC 0 min, median (IQR)	17.6 (10.4, 24.9)	10.2 (6.3, 13.8)	0.120
PAC 20 min, median (IQR)	55.3 (37.6, 90.4)	24.9 (18.8, 33.6)	< 0.005
PAC 40 min, median (IQR)	50.9 (44.3, 97.2)	26.1 (18.6, 33.3)	< 0.005
Cortisol 0 min, median (IQR)	8.1 (7.2, 9.7)	11.2 (9.2, 14.6)	0.010
Cortisol 20 min, median (IQR)	18.6 (17, 21.7)	21.2 (19.4, 24)	0.104
Cortisol 40 min, median (IQR)	21.7 (18.7, 24)	23.2 (20.9, 26)	0.304
Maximum PAC, median (IQR)	58.0 (54.1, 97.2)	26.8 (20.3, 35.2)	0.014
Maximum cortisol, median (IQR)	21.7 (18.7, 24)	23.5 (21, 26)	0.064
Maximum PAC/cortisol, median (IQR)	3.8 (1.9, 5.1)	1.1 (0.8, 1.5)	< 0.005
20-minute delta PAC	39.8 (27.2, 43.9)	14.4 (11.1, 19.8)	< 0.005
40-minute delta PAC	39.5 (22.0, 60.2)	15.0 (10.1, 19.4)	< 0.005

SD: Standard deviation, IQR: Interquartile range, PAC: Plasma aldosterone concentration, PRA: plasma renin activity, PAC unit: ng/dL, PRA unit: ng/mL/hr, serum cortisol unit: µg/dL

### Univariable and multivariable model of plasma aldosterone concentration after 1 µg ACTH stimulation test

Univariable analysis of the association between the PAC after the ACTH stimulation test and a diagnosis of PA revealed that all values of PAC (PAC at 20 and 40 min, maximum PAC, maximum PAC/cortisol, 20- and 40-minute delta PAC) were significantly associated with PA. For multivariable analysis adjusted for age, sex and BMI, only PAC at 20 min after the ACTH stimulation tests showed a significant association with a diagnosis of PA (*p* = 0.040). The data are shown in Table 2.

**Table 2:** Univariable and multivariable model of plasma aldosterone concentration after 1 µg ACTH stimulation test

Value	Univariable		Multivariable*	
	OR (95%CI)	p-value	OR (95% CI)	p-value
PAC 20 min	1.14 (1.03, 1.27)	0.010	**1.18 (0.99, 1.31)**	**0.040**
PAC 40 min	1.25 (1.02, 1.54)	0.029	1.50 (0.85, 2.66)	0.157
Maximum PAC	1.24 (1.03, 1.49)	0.019	1.33 (0.92, 1.93)	0.133
Maximum PAC/cortisol	2.04 (1.10, 3.79)	0.023	1.54 (0.82, 2.89)	0.177
20-minute delta PAC	1.41 (1.02, 1.95)	0.037	1.59 (0.95, 2.67)	0.079
40-minute delta PAC	1.21 (1.04, 1.41)	0.012	1.51 (0.74, 3.08)	0.254

PAC: Plasma aldosterone concentration, PRA: plasma renin activity

*Multivariable analysis was adjusted for age, sex and body mass index

### Diagnostic accuracy of plasma aldosterone concentration at 20 min after ACTH stimulation test

**Fig. 2:**
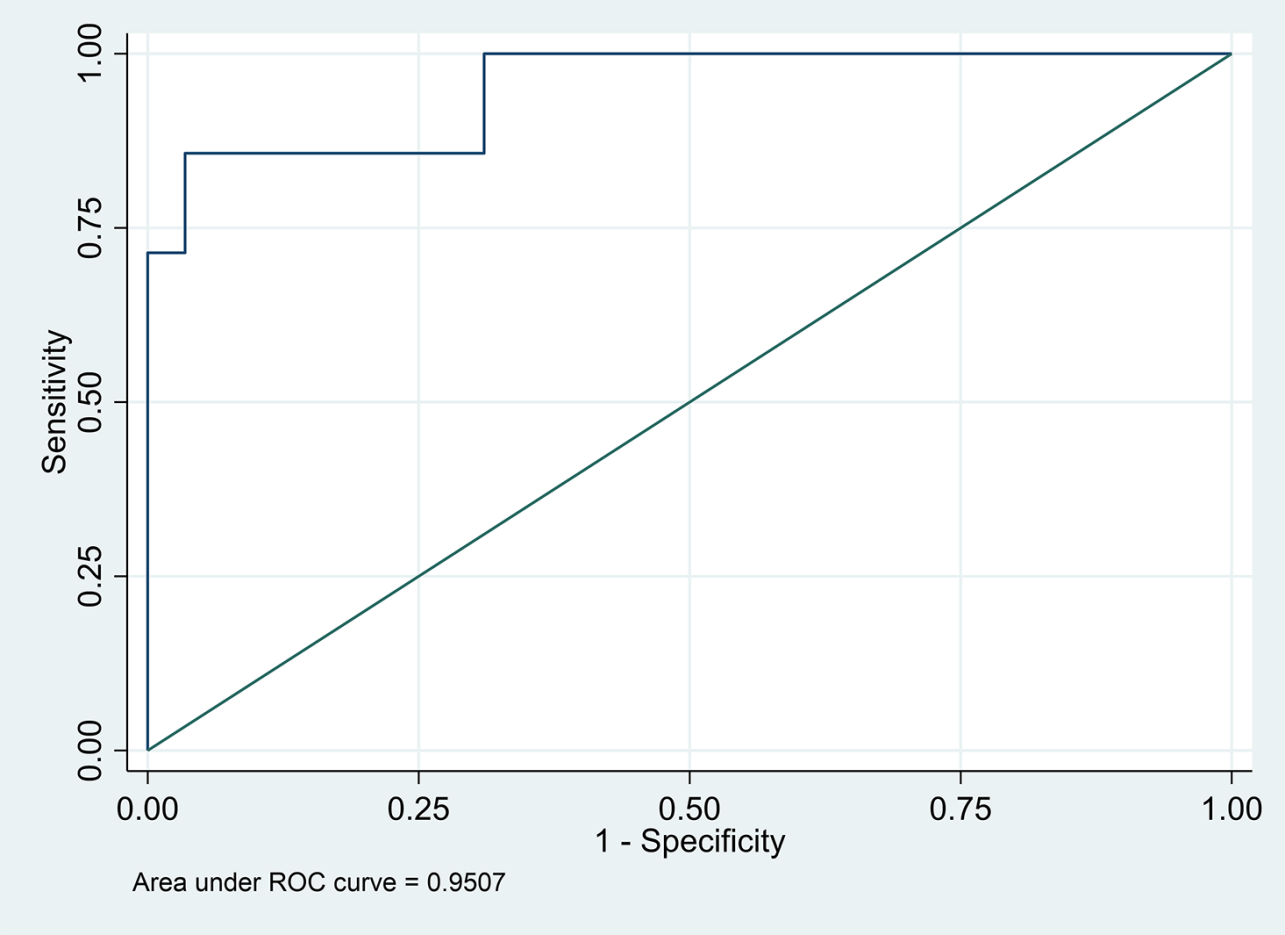
Standards for reporting of diagnostic accuracy (STARD) flow diagram of the study

The AuROC of PAC at 20 min after ACTH stimulation tests was 0.95 (Fig. 2). The optimal cut-off for PAC at 20 min was 52 ng/dL with a sensitivity of 71.4% with 95%CI (29, 96.3%) and a specificity 96.6% with 95%CI (82.2, 99.9). The positive likelihood ratio was 20.7 with 95%CI (2.8, 150.3) and the negative likelihood ratio was 0.3 with 95%CI (0.09,0.96).

## Discussion

The present study highlights the important finding that the PAC level 20 min after a 1 µg ACTH stimulation test has a significant association with the occurrence of PA. Also, the appropriate cut-off level of PAC at 20 min after ACTH stimulation test was demonstrated. This diagnostic procedure can help determine the presence of PA, which can potentially allow bypassing multistep investigations, particularly in patients who have contraindications to performing those confirmation tests, and can help in institutions where conducting confirmation tests is not possible.

Results of the use of the PAC level after ACTH stimulation test to determine PA status have been reported in multiple studies, most of which were performed in Japan. Those studies, which used PAC following the ACTH stimulation test to differentiate between APA and bilateral adrenal hyperplasia (BAH), showed high predictive performance based on AuROC and provided consistent results [7, 9, 18]. In contrast to predicting PA subtypes, the evidence regarding using ACTH stimulation tests for PAC to help diagnose PA is limited and has shown inconsistent results. To the best of our knowledge, two studies have reported that PAC after an ACTH stimulation test had no utility in the confirmation of PA [9, 10].

There has been only one study that, similar to the present study, demonstrated a positive association between PAC after ACTH stimulation and the confirmation of PA. That retrospective study by Inoue et al., which included 48 patients, reported that PAC at 30 min after a 250 µg ACTH stimulation test without 1-mg dexamethasone suppression had a high diagnostic accuracy for PA with an AuROC of 0.78 [11]. Their proposed cut-off for PAC at 30 min after an ACTH stimulation test was at 29.1 ng/dL with a sensitivity of 73.3% and a specificity of 66.7%. There were, however, some differences between their study and ours. First, our study used low dose (1 µg) ACTH rather than 250 µg. As the usual post-stimulation ACTH level in normal individuals is approximately 4,000 pg/mL, the use of a 1 µg ACTH stimulation is more physiologic than 250 µg [19]. Second, the PACs were drawn at 20 and 40 min after ACTH stimulation in our study while in the Inoue study, PAC was collected only at 30 min. A study by Honour et al. reported that after a low dose ACTH stimulation test the peak aldosterone response usually occurs 30 min after the test [12]. Thus, repeated measurement of PAC levels at intervals as in our study may more accurately detect peak levels of PAC. Additionally, the proposed cut-off for PAC in our study was higher than in the Inoue study, which could be explained by the different time points at which PAC levels were measured.

One possible reason for the conflicting results of ACTH stimulation testing for PAC level in the aforementioned studies is that those studies were based on small sample sizes, were single-center studies, and most were performed in one country, Japan. Further studies with larger samples and conducted in multiple countries are warranted to prove the concept that the ACTH stimulation test for PAC can be employed, allowing some invasive confirmation procedures for PA to be bypassed.

Strengths of the present study included, first, that the confirmation test for PA was performed by seated SIT which has higher sensitivity than recumbent SIT and that it had a lower proportion of inconclusive results than the fludrocortisone suppression test [20]. Second, the low dose ACTH used in this study better mimics a physiologic adrenal response than the high dose used in other studies. Lastly, the present study was one of the very few studies about ACTH stimulation tests and PA which conducted outside Japan.

There are several limitations in this study. First, internal validation could not be performed and the generalizability of this study was questionable due to the small sample size as this study was a preliminary study, performed in a single center and the small event rate of PA. Additional studies should be conducted with larger numbers of patients and with cohorts from different countries. Second, performing the dexamethasone suppression test prior to the ACTH stimulation test for PA is still controversial. In our study only ACTH stimulation without the dexamethasone suppression test was used. Third, the present study did not determine the subtypes of PA by using adrenal venous sampling or by CT adrenal gland, so only a diagnosis of PA or non-PA was reported. Subtypes of PA may affect the results, e.g., one report stated that aldosterone secretion in unilateral disease or APA patients is more likely to depend on endogenous ACTH than in patients with BAH [21]. Fourth, the PAC level measured by ACTH stimulation after unilateral adrenalectomy was not retrieved. Therefore, the result could not be further elucidated as to whether PAC was improved after surgery or not. Also, in this study, high dose ACTH stimulation tests (250 µg) and low dose ACTH stimulation tests (1 µg) were not compared. This study aimed to explore six different potential diagnostic indices. However, this approach may inadvertently inflate the statistical significance of the results by chance. Consequently, it is advisable to interpret the findings with caution. Lastly, the association of PAC level after saline suppression test and PAC level after ACTH stimulation test showed moderate to strong positive correlation. However, after multivariable regression analysis, there was no significant association between these two values (Supplementary appendix). Further study with the primary outcome of the association of PAC level after saline suppression test for PA confirmation and PAC level after ACTH stimulation test should be conducted.

## Conclusions

PAC level at 20 min after a 1 µg ACTH stimulation test may be used where confirmation testing for PA cannot be conducted. Further study of these results with larger samples and with internal and external validation is warranted.

### Electronic supplementary material

Below is the link to the electronic supplementary material.

Supplementary Material 1

Supplementary Material 2

## Data Availability

The data that support the findings of this study are available upon request from the authors.

## References

[1] Brown JM, Siddiqui M, Calhoun DA, Carey RM, Hopkins PN, Williams GH, Vaidya A (2020). The unrecognized prevalence of primary aldosteronism: a cross-sectional study. Ann Intern Med.

[2] Funder JW, Carey RM, Mantero F, Murad MH, Reincke M, Shibata H, Stowasser M, Young WF (2016). Jr: the management of primary aldosteronism: case detection, diagnosis, and treatment: an endocrine Society Clinical Practice Guideline. J Clin Endocrinol Metabolism.

[3] Mulatero P, Milan A, Fallo F, Regolisti G, Pizzolo F, Fardella C, Mosso L, Marafetti L, Veglio F, Maccario M (2006). Comparison of confirmatory tests for the diagnosis of primary aldosteronism. J Clin Endocrinol Metab.

[4] Nanba K, Tsuiki M, Umakoshi H, Nanba A, Hirokawa Y, Usui T, Tagami T, Shimatsu A, Suzuki T, Tanabe A (2015). Shortened saline infusion test for subtype prediction in primary aldosteronism. Endocrine.

[5] Hattangady NG, Olala LO, Bollag WB, Rainey WE (2012). Acute and chronic regulation of aldosterone production. Mol Cell Endocrinol.

[6] Kem DC, Weinberger MH, Higgins JR, Kramer NJ, Gomez-Sanchez C, Holland OB (1978). Plasma aldosterone response to ACTH in primary aldosteronism and in patients with low renin hypertension. J Clin Endocrinol Metab.

[7] Sonoyama T, Sone M, Miyashita K, Tamura N, Yamahara K, Park K, Oyamada N, Taura D, Inuzuka M, Kojima K (2011). Significance of adrenocorticotropin stimulation test in the diagnosis of an aldosterone-producing adenoma. J Clin Endocrinol Metab.

[8] Moriya A, Yamamoto M, Kobayashi S, Nagamine T, Takeichi-Hattori N, Nagao M, Harada T, Tanimura-Inagaki K, Onozawa S, Murata S (2017). ACTH stimulation test and computed tomography are useful for differentiating the subtype of primary aldosteronism. Endocr J.

[9] Umakoshi H, Xiaomei Y, Ichijo T, Kamemura K, Matsuda Y, Fujii Y, Kai T, Fukuoka T, Sakamoto R, Ogo A (2017). Reassessment of the cosyntropin stimulation test in the confirmatory diagnosis and subtype classification of primary aldosteronism. Clin Endocrinol (Oxf).

[10] Terui K, Kageyama K, Nigawara T, Moriyama T, Sakihara S, Takayasu S, Tsushima Y, Watanki Y, Yamagata S, Sugiyama A (2016). Evaluation of the (1–24) adrenocorticotropin stimulation test for the diagnosis of primary aldosteronism. J Renin Angiotensin Aldosterone Syst.

[11] Inoue K, Omura M, Sugisawa C, Tsurutani Y, Saito J, Nishikawa T. Clinical utility of the Adrenocorticotropin Stimulation Test with/without Dexamethasone suppression for definitive and subtype diagnosis of primary Aldosteronism. Int J Mol Sci 2017, 18(5). 10.3390/ijms18050948 PMC5454861 28468286

[12] Honour JW, Bridges NA, Conway-Phillips E, Hindmarsh PC (2008). Plasma aldosterone response to the low-dose adrenocorticotrophin (ACTH 1–24) stimulation test. Clin Endocrinol (Oxf).

[13] Mancini T, Kola B, Mantero F, Arnaldi G (2003). Functional and nonfunctional adrenocortical tumors demonstrate a high responsiveness to Low-Dose Adrenocorticotropin. J Clin Endocrinol Metabolism.

[14] Rossi GP, Bisogni V, Bacca AV, Belfiore A, Cesari M, Concistrè A, Del Pinto R, Fabris B, Fallo F, Fava C (2020). The 2020 Italian society of arterial hypertension (SIIA) practical guidelines for the management of primary aldosteronism. Int J Cardiol Hypertens.

[15] Bornstein SR, Allolio B, Arlt W, Barthel A, Don-Wauchope A, Hammer GD, Husebye ES, Merke DP, Murad MH, Stratakis CA (2016). Diagnosis and treatment of primary adrenal insufficiency: an endocrine Society Clinical Practice Guideline. J Clin Endocrinol Metab.

[16] Anantharaman R, Menezes G, Yusuf R, Ganapathi B, Ayyar SV, Srinivasan R (2013). The 1 µg cosyntropin test in normal individuals: a reappraisal. Indian J Endocrinol Metab.

[17] Liu X (2012). Classification accuracy and cut point selection. Stat Med.

[18] Jiang Y, Zhang C, Wang W, Su T, Zhou W, Jiang L, Zhu W, Xie J, Ning G (2015). Diagnostic value of ACTH stimulation test in determining the subtypes of primary aldosteronism. J Clin Endocrinol Metab.

[19] Peeters B, Meersseman P, Vander Perre S, Wouters PJ, Debaveye Y, Langouche L, Van den Berghe G (2018). ACTH and cortisol responses to CRH in acute, subacute, and prolonged critical illness: a randomized, double-blind, placebo-controlled, crossover cohort study. Intensive Care Med.

[20] Stowasser M, Ahmed A, Cowley D, Wolley M, McWhinney B, Ungerer J, Gordon RD. SEATED SALINE SUPPRESSION TESTING FOR THE DIAGNOSIS OF PRIMARY ALDOSTERONISM. A VALIDATION STUDY. J Hypertens 2018, 36. 10.1210/jc.2018-01394 30239841

[21] Sonoyama T, Sone M, Tamura N, Honda K, Taura D, Kojima K, Fukuda Y, Kanamoto N, Miura M, Yasoda A (2014). Role of endogenous ACTH on circadian aldosterone rhythm in patients with primary aldosteronism. Endocr Connect.

