# Supplementary material for: Plasma aldosterone response to ACTH stimulation test for diagnosis of primary aldosteronism: a cross-sectional study
Source: BMC Endocr Disord. 2024 Mar 13;24:37. doi: 10.1186/s12902-024-01563-y (PMC10935999; doi:10.1186/s12902-024-01563-y)
Supplement: Supplementary file 1 — Supplementary Material 1 [file 12902_2024_1563_MOESM1_ESM.docx]

**Supplementary appendix**

**Table S1** The correlation of PAC level after ACTH stimulation test and PAC level after saline infusion test

|  | **Correlation (*r*) with PAC after NSS infusion** |
| --- | --- |
| **PAC at 20 min** | 0.70 |
| **PAC at 40 min** | 0.69 |

**Table S2** Multivariable analysis of PAC level after ACTH stimulation test and PAC level after saline infusion test

|  | **Coefficient** | **p-value** |
| --- | --- | --- |
| **PAC at 20 min** | 3.44 | 0.150 |
| **PAC at 40 min** | 1.83 | 0.069 |
